# The follow-up of preterm infant after discharge: family pediatrician (FP) medical viewpoint

**DOI:** 10.1186/1824-7288-41-S2-A34

**Published:** 2015-09-30

**Authors:** Silvia Gambotto, Marina Picca

**Affiliations:** 1 family pediatrician ASL 4 Turin, Italy -SICuPP; 2 family pediatrician ASL Milan, Italy - SICuPP

## 

Survival rates and outcomes of infant and preterm infants have radically improved thanks to the most recent techniques of resuscitation and intensive care. Premature babies are exposed to an higher risk of growth problems, delayed development or complex medical problems and, compared to other children, have a higher prevalence of severe disability [1].

In order to discharge premature infants from neonatal intensive care we have to consider some critical aspects [2]. The active and early involvement of family pediatrician (FP) is essential to ensure therapeutic success . These infants represent a small portion of births (0.3 % among the choices of a FP), but they're becoming more frequent . The definition of an individualized and shared assistance and follow-up program requires the establishment of an effective flow of information that encourages a two-way path of information and patients . In low complexity and risk cases, child and family should be assigned to a team consisting of FP, child psychiatrist and other health professionists for rehabilitation in residence territory.

The primary objective is to promote especially the early management of infants with gestational age less than 28 weeks, discharged from neonatal intensive care, favoring, before being discharged from the hospital, family involvement, integration between hospital professionists and local services involved in determination and implementation of an Individual Support Plan (IAP), in order to follow the child in his first year of life.

FP during the specific and agreed health statements can evaluate and closely monitor growth, development, neuro developmental, visual and hearing functions and any medical problems still unresolved. The preterm infant must be subjected, according to the chronological age, to all recommended vaccinations, considering too the influenza vaccination. The FP figure is crucial to promote, encourage and support vaccination and preventive route (SRV).

FP must improve specific skills to be able to welcome and assist the premature infant and his family avoiding their unnecessary trips to hospitals. It is also important to provide intra and interprofessional training processes to promote team work.

A real integration between different levels of care and a definition of roles and professional tasks, might avoid efforts duplication and ensure continuity of care, reducing discomfort and uncertainty of families.
